# Immune Checkpoint Blockade for Aspergillosis and Mucormycosis Coinfection

**DOI:** 10.1097/HS9.0000000000000530

**Published:** 2021-02-10

**Authors:** Jan Christoph Banck, Niklas Mueller, Sibylle Christiane Mellinghoff, Martin Thelen, Alessia Fraccaroli, Viktoria Blumenberg, Philipp Koehler, Wolfgang Gerhard Kunz, Martina Rudelius, Florian Schrötzlmair, Marion Subklewe, Hans Anton Schlößer, Johanna Tischer, Oliver Andreas Cornely, Lars Hartwin Lindner, Michael von Bergwelt-Baildon

**Affiliations:** 1 Department of Medicine III, University Hospital, LMU Munich, Germany; 2 Laboratory for Translational Cancer Immunology, Gene Center, LMU Munich, Germany; 3 University of Cologne, Faculty of Medicine and University Hospital Cologne, Department I of Internal Medicine, Center for Integrated Oncology Aachen Bonn Cologne Duesseldorf (CIO ABCD), Excellence Center for Medical Mycology (ECMM), Cologne, Germany; 4 University of Cologne, Cologne Excellence Cluster on Cellular Stress Responses in Aging-Associated Diseases (CECAD), Cologne, Germany; 5 Center for Molecular Medicine Cologne, University of Cologne, Faculty of Medicine and University Hospital Cologne, Germany; 6 Department of Radiology, University Hospital, LMU Munich, Germany; 7 Institute of Pathology, Ludwig-Maximilians-University Munich, Germany; 8 Department of Otorhinolaryngology, Head & Neck Surgery, University Hospital, LMU Munich, Germany; 9 Department of General, Visceral, Cancer and Transplantation Surgery, University of Cologne, Faculty of Medicine and University Hospital Cologne, Germany; 10 Clinical Trials Centre Cologne (ZKS Köln), University of Cologne, Germany; 11 German Cancer Consortium (DKTK), partner site Munich, Germany; 12 Intensive Care in Hematologic and Oncologic Patients (iCHOP), Cologne, Germany; 13 Comprehensive Cancer Center Munich (CCCM), Germany.

Immunocompromised patients are exposed to a heightened risk of invasive fungal infections (IFIs) such as aspergillosis and mucormycosis causing substantial morbidity and mortality.^1,2^ Surgical debridement along with systemic antifungal drugs is the current treatment strategy,^2^ but the prognosis of these patients is still very poor.^1,2^ Therefore, new therapeutic approaches are urgently needed. Recently published in vitro and in vivo studies imply a benefit of checkpoint inhibition for salvage treatment of IFI.^3-7^ Here, we report the first immunosuppressed hematological patient receiving the immune checkpoint inhibitor nivolumab plus interferon gamma (IFN-γ) as salvage treatment for aspergillus and mucormycosis coinfection (Supplemental Figure 1, http://links.lww.com/HS/A132).

The patient, a 51-year-old woman with relapsed acute myeloid leukemia (AML) after human leukocyte antigen (HLA)-identical allogeneic hematopoietic stem cell transplantation (HSCT), was diagnosed with AML without maturation (French-American-British M1) in May 2015 and had an adverse risk constellation according to European Leukemia Net classification. Molecular genetic testing revealed fms-like tyrosine kinase 3 length (ratio 0.165), runt-related transcription factor 1, and BCL6 corepressor mutations. Cytogenetically, a 47 X-chromosome +14 karyotype was found. After inclusion in the Dauno-Double study (NCT02140242), she was treated with a single course of “7 + 3” induction chemotherapy followed by high-dose cytarabine consolidation and achieved complete remission. In August 2018, after conditioning treatment with fludarabine-8Gy total body irradiation, she received allogeneic HSCT (donor HLA-identical brother). Four months later, a relapse with decreasing donor chimerism (77%) was diagnosed. During donor search for a second HSCT, the patient received azacitidine and lenalidomide from March to June 2019 as a bridging treatment in analogy to AZALENA study (NCT02472691), but without response. No anti-mold prophylaxis was administered. In June 2019, the patient presented with fever and sinusitis complaints. At that time, the patient was in pancytopenia (leukocytes 3.23 G/L, neutrophils 0.19 G/L, platelets 20 G/L, hemoglobin 9.80 g/dL) with relative lymphocytosis (74%). Inflammation parameters were elevated. A computed tomography (CT) scan exhibited pansinusitis with obstruction of ethmoid, sphenoid, and maxillary sinuses (Figure 1A); no pulmonary lesions were seen. *Lichtheimia ramosa* and *Aspergillus fumigatus* were detected microbiologically, both susceptible to amphotericin B, isavuconazole, and posaconazole. Ethmoidectomy was performed and intravenous treatment with liposomal amphotericin B (L-AmB 5 mg/kg intravenous [IV]/d) was initiated. Invasive mucormycosis was confirmed by immunohistochemistry (Grocott and periodic acid–Schiff-positive hyphae). A follow-up CT scan after 3 days revealed progression of mucormycosis (Figure 1B). Several pansinus revisions with bilateral median hemimaxillectomy and subtotal resection of the necrotic nasal septum were executed (Figure 1C). Excessive bleeding demanded repetitive packing. Intravenous treatment with L-AmB (10 mg/kg IV/d) was dose-escalated and complemented by isavuconazole (200 mg IV/d following loading dose of 200 mg thrice/d for 2 d). In addition, 300 μg filgrastim was administered daily for 25 days without effect. Despite this multimodal approach, mucormycosis progressed with bone destruction and spread to both orbits (Figure 1D).

**Figure 1.**
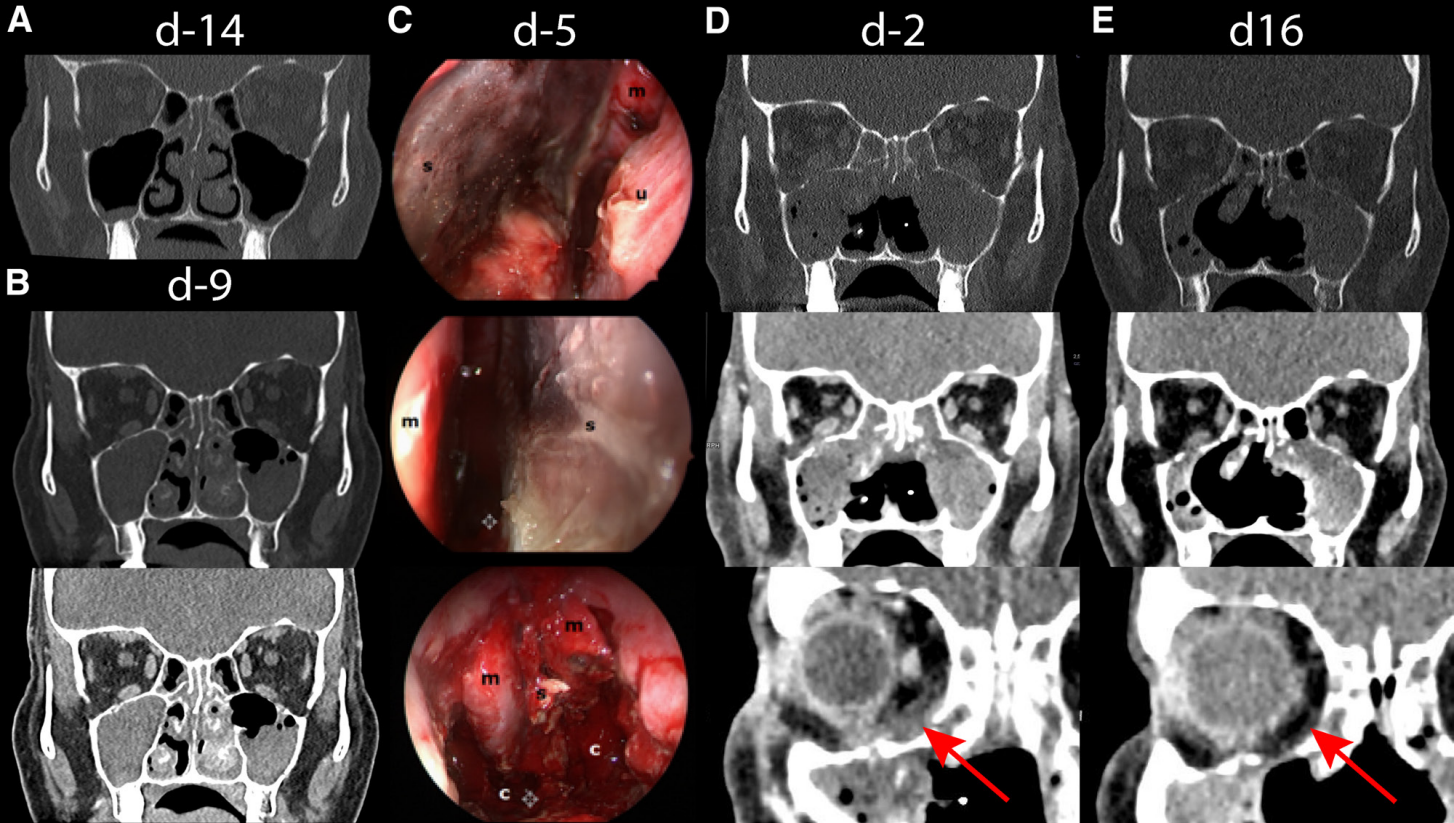
**CT scans and intraoperative images.** Initial CT scan showing chronic infection of the paranasal sinuses with obstruction of ethmoidal, sphenoidal, and maxillary sinuses 1 d prior to diagnosis of mixed invasive fungal infection with aspergillosis and mucormycosis on day –13 (A). A follow-up CT scan (3 d after classical endonasal ethmoidectomy on day –9) revealed progression of paranasal mucormycosis (B). Several pansinus revisions with median hemimaxillectomy on both sides and subtotal resection of the necrotic nasal septum were performed. The intraoperative images on day –5 (C) show the necrotic nasal septum (s) from both sides and the status after its resection. A postoperative CT scan on day –2 revealed a further progression of paranasal mucormycosis with increased bone affection of the paranasal sinuses and new bilateral infiltration of the orbit (D). Sixteen days after treatment initiation with nivolumab on day 0 complemented by 5 doses of interferon-γ, a CT scan revealed a partial remission with regression of mucormycosis invasion in the sphenoidal and maxillary sinuses (E). c = choanae; CT = computed tomography; m = middle turbinate; u = inferior turbinate.

Due to missing treatment response and patient’s refusal of radical surgery, salvage immune therapy was begun 12 days after initiation of antifungal treatment (day 0). Simultaneously to L-AmB 10 mg/kg and isavuconazole 200 mg daily, nivolumab 240 mg was administered IV and complemented by 5 doses of 100 μg IFN-γ (thrice weekly). Surgery was restricted to minimal interventions (debridement, hemostasis, change of packing) and intraoperative nasal/paranasal rinsing with 500 mg L-AmB was performed once. Administration of nivolumab was repeated every 2 weeks (4 doses in total). IFN-γ was stopped after 10 doses because of recurrent fever. From day 10, no further surgery was performed and local swelling disappeared. Olfaction and laboratory inflammatory markers improved. A CT scan on day 16 revealed partial response of mucormycosis (Figure 1E). On day 20 L-AmB dose was halved and on day 28 further reduced to 5 mg/kg thrice weekly, while isavuconazole 200 mg was continued. Follow-up CTs until day 40 showed stable disease. Nine weeks after diagnosis and 4 doses of nivolumab, the patient was discharged. A significant progression of AML was confirmed by bone marrow biopsy (with 60% myeloblasts, 36% donor chimerism). Continuing antifungals, IFI remained stable for another month. Then the patient developed fever, refused antibiotics and readmission, and died from septic shock with disseminated intravascular coagulation.

To characterize the patient’s lymphocytic immune response, blood samples were collected before treatment, 2 (under treatment [UT] 1) and 5 weeks (UT 2) after nivolumab initiation. Peripheral blood mononuclear cells were isolated and analyzed by flow cytometry. Using a defined panel of antibodies (Supplemental Table S1, http://links.lww.com/HS/A133), major lymphocyte subsets were further characterized by their expression of activation/differentiation markers and immune checkpoint molecules (Figure 2A–C). Under nivolumab treatment, CD86 and CD69 increased on B- and T-cells indicating a systemic immune activation.^8,9^ The percentage of memory T-cells remained stable, the percentages of memory B-cells and plasmablasts increased. The expression of programmed cell death 1 (PD-1) and cytotoxic T-lymphocyte-associated protein 4 substantially decreased. The same applies to CD39, lymphocyte-activation gene 3, and programmed death ligand 1 (PD-L1), although they initially showed an increased expression (UT1). T-cell immunoglobulin and mucin-domain containing-3 and the co-stimulatory molecules OX40 and CD40L showed at first an increased expression and remained stable in the further course. DNAX accessory molecule-1 was found to be generally high expressed and stable under therapy. Immunohistochemical staining of the resected ethmoid bone and nasal septum revealed Grocott positive hyphae within small vessels surrounded by PD-1 and PD-L1 positive cells (Figure 2D).

**Figure 2.**
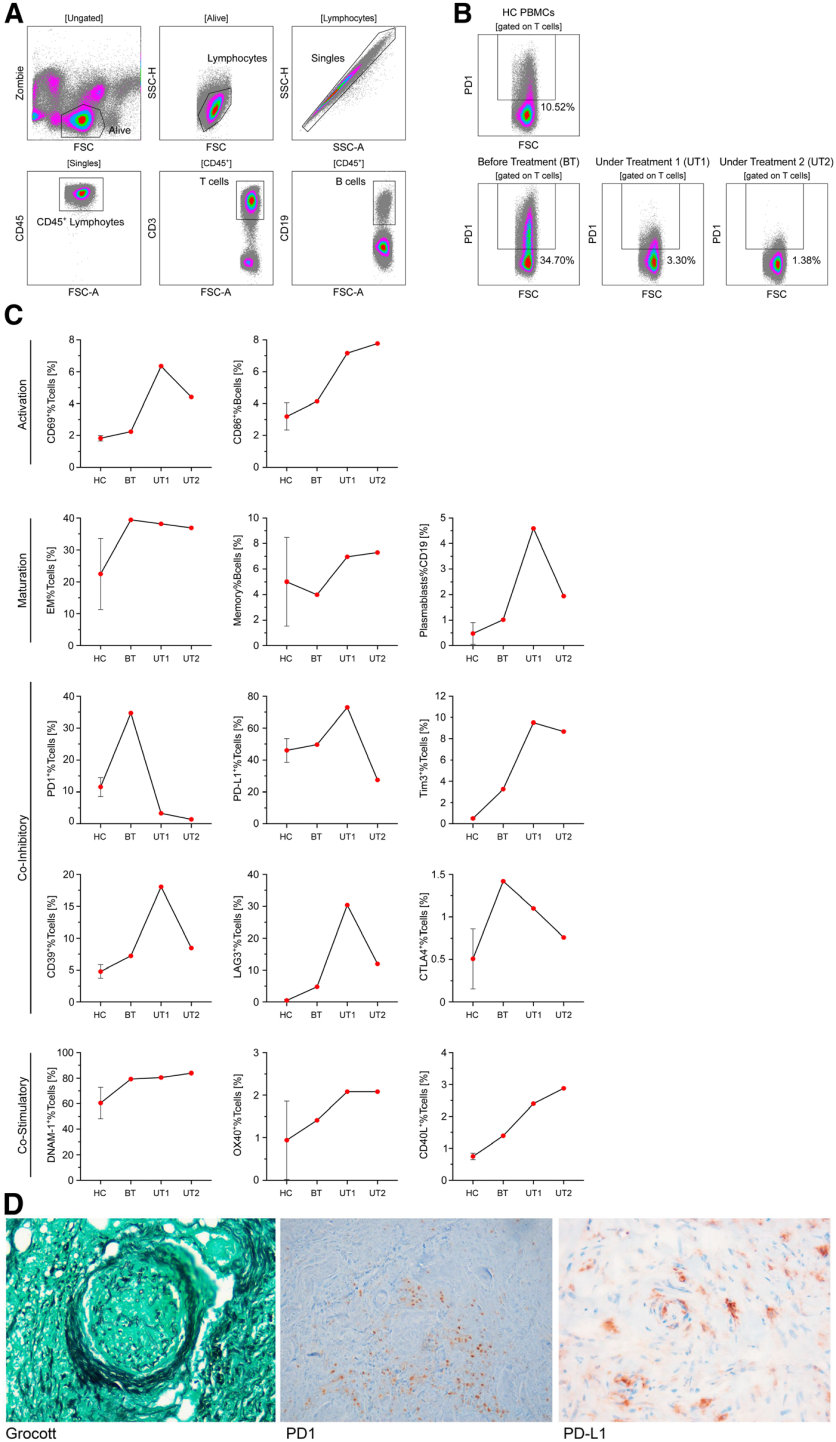
**Flow cytometry and histopathological analyses.** (A and B), Detailed gating strategy. (B), PD-1 expression on T-cells of HC PBMCs and PBMCs BT and UT with nivolumab (timepoint number 1 = UT1 and timepoint number 2 = UT2). (C), Expression of indicated markers on T-cells of HC PBMCs (n = 3) and patterns of PBMCs BT, 2 wk (UT1) and 5 wk (UT2) after nivolumab initiation. (D), Immunohistochemical Grocott staining of resected ethmoid bone and nasal septum demonstrating angioinvasion of mucor hyphae with grouping of PD-1 and PD-L1 positive cells around the mucor focus. BT = before treatment; CTLA4 =cytotoxic T-lymphocyte-associated protein 4; DNAM-1 = DNAX accessory molecule-1; EM = effector memory; FSC = forward scatter; FSC-A = forward scatter area; HC = healthy control; LAG3 = lymphocyte-activation gene 3; PBMC = peripheral blood mononuclear cell; PD-1 = programmed cell death 1; PD-L1 = programmed death ligand 1; SSC-A = side scatter area; SSC-H = side scatter height; TIM-3 = T-cell immunoglobulin and mucin-domain containing-3; UT = under treatment.

Immune checkpoints inhibit T-cell function, thus controlling immune response by preventing excessive reaction.^10,11^ Nivolumab, a monoclonal antibody, targets PD1. PD-1 is expressed on activated lymphocytes, monocytes, and natural killer cells, whereas its ligand PD-L1 is expressed on antigen-presenting cells and on the surface of nonhematopoietic cells including tumor cells.^12^ Blockade of the PD-1/-PD-L1-axis by immune checkpoint inhibitors as nivolumab has been shown to restore immunity and is used to treat several malignancies with great success.^13^ While IFN-γ was shown to restore immunity in patients with IFI,^14^ recently published in vitro and in vivo studies imply a probable benefit of checkpoint inhibition for salvage treatment of bacterial and IFIs.^3,5,7^ Patients with candidemia showed an immune phenotype consistent with T-cell exhaustion and downregulation of co-stimulatory markers.^3^ To date, only 1 young polytrauma patient with abdominal mucormycosis recovered after nivolumab treatment plus IFN-γ.^5^ In our immunocompromised hematological patient with mixed IFI, nivolumab complemented by IFN-γ led to clinical response with significant improvement of the patient’s condition. By blocking the PD-1/PD-L1-axis, nivolumab reduced patient’s immunosuppression and infectious symptoms despite progression of the underlying AML. Compared with healthy control, the percentage of T-cells expressing PD-1 increased substantially and declined following administration of nivolumab indicating target saturation.^15^ Histological staining showing angioinvasion of mucor hyphae with grouping of PD-1 and PD-L1 positive cells around the mucor foci supports our hypothesis of immunosuppression within our patient’s infectious microenvironment. The effect of nivolumab on the underlying AML remains unclear. According to current data, checkpoint inhibitors apparently only have limited effect in AML patients.^16^ Concerning the mechanism of action in patients with IFI, we assume that nivolumab targets their lack of immune defense through PD-1 by restoring T-cell function and therefore strengthening host immunity. Targeting immune checkpoints and thereby reversing hyporesponsiveness of the adaptive immune system seems to be beneficial in patients with IFI and should be determined in forthcoming studies.

The online version of this article contains a data supplement.

## Disclosures

SCM was a consultant to Octapharma; she has been receiving research grants from the University Hospital of Cologne (KoelnFortune), from the German Center for Infection Research (DZIF; Clinical Leave Stipend), and from the German Mycological Society (Dr Manfred Plepmpel Stipend). OAC is supported by the German Federal Ministry of Research and Education; he is funded by the Deutsche Forschungsgemeinschaft (DFG, German Research Foundation) under Germany’s Excellence Strategy—Cluster of Excellence Cellular Stress Responses in Aging-Associated Diseases, EXC 2030—390661388 and has received research grants from Actelion, Amplyx, Astellas, Basilea, Cidara, Da Volterra, F2G, Gilead, Janssen Pharmaceuticals, Medicines Company, MedPace, Melinta Therapeutics, Merck/Merck, Sharp & Dohme (MSD), Pfizer, Scynexis; he is a consultant to Actelion, Allecra Therapeutics, Al-Jazeera Pharmaceuticals, Amplyx, Astellas, Basilea, Biosys UK Limited, Cidara, Da Volterra, Entasis, F2G, Gilead, Matinas, MedPace, Menarini Ricerche, Merck/MSD, Mylan Pharmaceuticals, Nabriva Therapeutics, Octapharma, Paratek Pharmaceuticals, Pfizer, PSI, Rempex, Roche Diagnostics Scynexis, Seres Therapeutics, Tetraphase, Vical; and he received lecture honoraria from Astellas, Basilea, Gilead, Grupo Biotoscana, Merck/MSD, and Pfizer. MvBB has been a consultant to MSD Sharp & Dohme, Novartis, Roche, KITE/Gilead, Bristol-Myers Squibb, Astellas, Mologen, and Miltenyi; he has received research funding and honoraria from the aforementioned companies. The remaining authors have no conflicts of interest to disclose.

## Acknowledgments

We thank Eleni Lapsanidou for organizational support.

## Supplementary Material


