# Supplementary material for: Immune Checkpoint Blockade for Aspergillosis and Mucormycosis Coinfection
Source: Hemasphere. 2021 Feb 10;5(3):e530. doi: 10.1097/HS9.0000000000000530 (PMC7886469; doi:10.1097/HS9.0000000000000530)
Supplement: Supplementary file 1 [file hs9-5-e530-s001.pdf]

Supplemental Figure 1

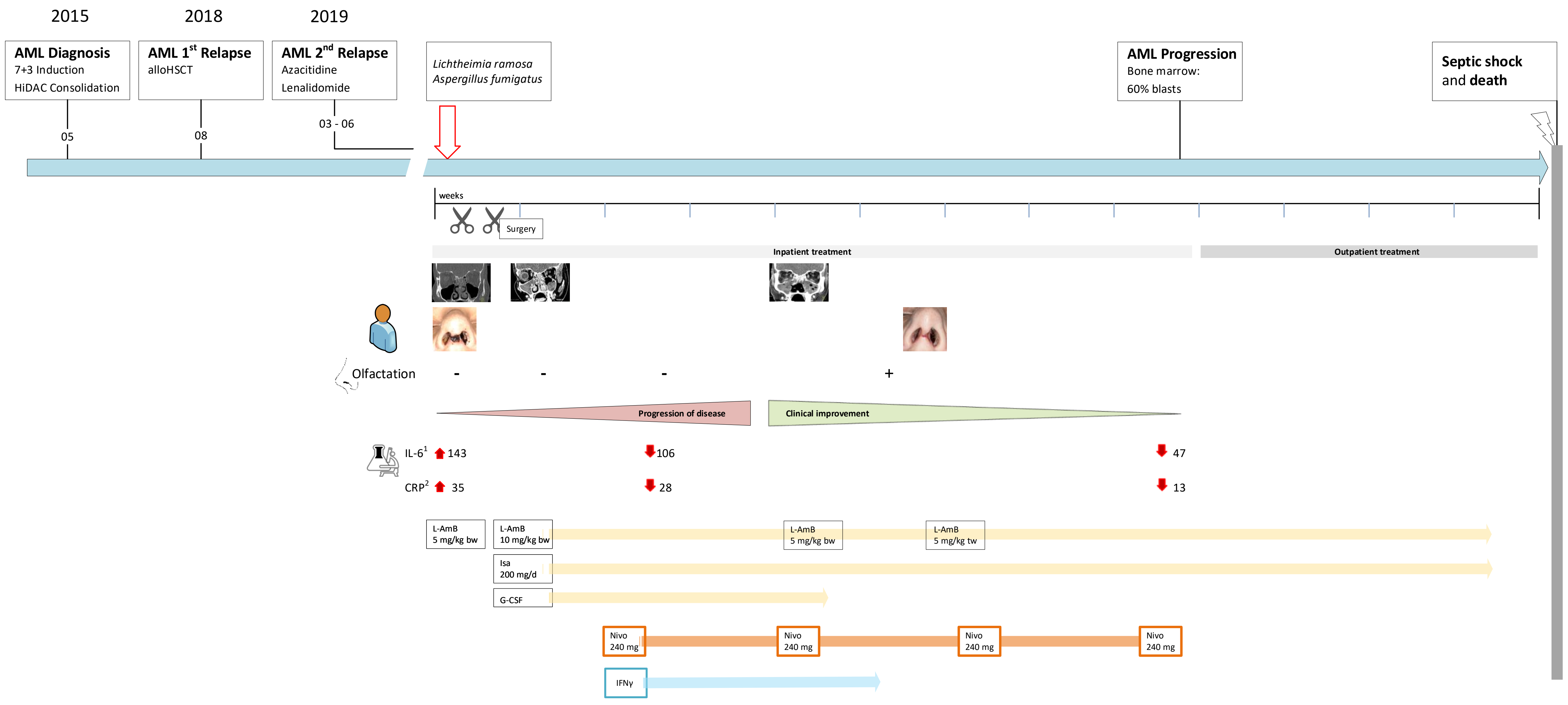

Figure S1. Chronological overview.
Patient's clinical course is shown from initial diagnosis of AML followed by two relapses and finally co-infection of invasive aspergillosis and mucormycosis. The overview displays the timetable of the multimodal treatment concept, diagnostics and patient's symptoms.
Abbreviations: HiDAC=High Dose Ara-C, IL-6=interleukin-6, CRP=c-reactive protein, mg=milligram, kg=kilogram, LAmB=Liposomal Amphotericin B, Nivo=Nivolumab, ISA=isavuconazol, G-CSF=granulocyte-colony stimulating factor, IFN-γ=Interferon-γ
1 [pg/ml], 2 [mg/dl].
