# Supplementary material for: Immune Checkpoint Blockade for Aspergillosis and Mucormycosis Coinfection
Source: Hemasphere. 2021 Feb 10;5(3):e530. doi: 10.1097/HS9.0000000000000530 (PMC7886469; doi:10.1097/HS9.0000000000000530)
Supplement: Supplementary file 2 [file hs9-5-e530-s002.pdf]

Flow Cytometry Antibodies				
**Fluorochrome**	**Target**	**Clone**	**Company**	**Catalogue**
FITC	CD20	2H7	Biolegend	302304
PerCP Cy5.5	CD38	HIT2	Biolegend	303522
PE	CD86	IT2.2	Biolegend	305406
PE	CCR7	G043H7	Biolegend	353204
PE	CTLA4	BNI3	BD	555853
PE-Dazzle 594	IgD	IA6-2	Biolegend	348240
PE-Dazzle 594	Tim3	F38-2E2	Biolegend	345034
PE-Dazzle 594	OX40	Ber-ACT35	Biolegend	350020
PE-Dazzle 594	CD40L	11C3.1	Biolegend	326308
PE-Cy7	CD45RA	HI100	Biolegend	304126
PE-Cy7	CD27	O323	Biolegend	302838
AF700	CD3	SK7	Biolegend	344822
AF647	PD1	EH12.1	BD	560838
APC	CD226 (DNAM-1)	11A8	Biolegend	338312
BV421	PD-L1	29E.2A3	Biolegend	329714
BV421	CD39	A1	Biolegend	328214
BV421	LAG3	11C3C65	Biolegend	369314
BV605	CD69	FN50	Biolegend	310937
BV785	CD45	HI30	Biolegend	304048
BUV737	CD19	SJ25C1	BD	564303
Zombie UV	fixable viability dye		Biolegend	423108
